# The Motility of β-Cyclodextrins Threaded on the Polyrotaxane Based Triblock Polymer and Its Influences on Mechanical Properties

**DOI:** 10.3390/ma17153757

**Published:** 2024-07-30

**Authors:** Yufei Wang, Yafang Niu, Xue Geng, Zengguo Feng, Lin Ye

**Affiliations:** 1 Tangshan Research Institute, Beijing Institute of Technology, Tangshan 063000, China; 3220235373@bit.edu.cn (Y.W.); 3220231536@bit.edu.cn (Y.N.); 2 School of Materials Science and Engineering, Beijing Institute of Technology, Beijing 100081, China; 7420121006@bit.edu.cn (X.G.); sainfeng@bit.edu.cn (Z.F.)

**Keywords:** polyrotaxane, molecular motility, β-CD, dielectric, mechanical property

## Abstract

Polyrotaxane (PR) has garnered increasing attention due to its unique structure and exceptional performance. In this study, a polypseudorotaxane (PPR) initiator was prepared through the self-assembly of bromine-capped Pluronic F68 and β-cyclodextrins (β-CD) in water. Polyrotaxane-containing triblock copolymers (PR copolymers) were successfully synthesized by atom transfer radical polymerization (ATRP) of butyl methacrylate (BMA) using the PPR initiator in the presence of Cu(I)Br/PMDETA. The structure of the PR copolymers was thoroughly characterized using infrared spectroscopy (IR), proton nuclear magnetic resonance (^1^H NMR), and gel permeation chromatography (GPC). The mobility of β-CD in the PR copolymers was demonstrated through dielectric measurements. Mechanical tests, including tensile strength assessments, thermal mechanical analysis, and dynamic mechanical analysis, confirmed the excellent mechanical properties and good processability of the PR copolymer, attributed to the PBMA blocks. Furthermore, the mechanical properties were found to be modulated by the motility of the threaded β-CDs. Consequently, the superior mechanical properties and the high mobility of the threaded β-CDs suggest promising potential for the as-prepared PR polymer in various advanced applications.

## 1. Introduction

Polyrotaxane (PR) has garnered increasing attention in recent years due to its unique structure and excellent stimuli-responsive properties [1,2,3]. Typically, PR is formed through the self-assembly of cyclodextrins (CDs) and linear polymers in aqueous solutions, followed by an end-capping reaction [4,5]. The CDs are threaded onto the linear polymer via hydrophobic interactions instead of covalent bonds, allowing them to move along and rotate around the linear polymer freely. This unique motility is believed to confer numerous advantages to PR [6,7,8]. For example, Ito crosslinked the threaded CDs in the PR copolymer to create what is known as a “sliding gel” [9,10]. Unlike traditional chemically crosslinked gels, which have fixed crosslinking points, the sliding gel features sliding crosslinking points due to the motility of the CDs. This unique characteristic imparts a distinct J-shaped stress–strain curve and provides exceptional anti-scratch and self-healing properties to the sliding gel [11,12]. When stimuli-responsive (SR) groups are introduced into the threaded CDs, PR may exhibit better and faster SR behavior compared to traditional SR polymers. The rate of shape recovery (SR) behavior is related to the motility of the SR moieties. Since they are usually covalently bonded to the material’s backbone, their movement is restricted by neighboring moieties. This restriction is known as the “matrix effect”. Thus, the improvement in SR behavior by PR materials is due to the SR groups on the PR being free from the “matrix effect” restrictions, thanks to the excellent motility of the CDs [13,14]. Moreover, some researchers, including our team, have reported that dynamic ligands grafted onto the threaded CDs can effectively regulate cell adhesion [15,16], proliferation [17], and differentiation [18] through the high motility of the CDs. As a result, PR is recognized as a promising candidate for various functional materials applications [19,20].

However, similar to other supramolecules, most of PR’s smart functions have been observed in solution, which may significantly limit its applications. Compared to the solution state, solid materials offer unparalleled advantages as they can be processed into any shape and withstand harsh environmental conditions such as high temperature, heavy load, high stress, and low pressure. For example, in vascular tissue engineering, artificial blood vessel scaffolds need to withstand the periodic pressure of blood flow and possess high compliance with these pressure changes [21,22,23,24]. Solid PR materials, with their mobile threaded CDs and high mechanical properties, can meet these critical requirements. Furthermore, incorporating bioactive ligands into the mobile CDs to form dynamic ligands could enhance the rapid endothelialization of the artificial blood vessel scaffold. Our previous findings indicate that dynamic ligands can effectively regulate endothelial cell spreading and proliferation through the high motility of the CDs [17]. Consequently, developing solid PR materials for functional applications remains a significant challenge and expectation for materials scientists. In addition to the preparation of solid PR materials, another critical question persists: Does the threaded CD in solid PR materials exhibit similar motility to that in the solution state, given the differences in molecular conformation and chemical environment between solution and solid states? Ito and colleagues addressed this by compressing PR powders at 4.5 kbar to form pellets and demonstrated the motility of CDs in the solid state through dielectric spectra of the pellet samples [25,26,27]. This strongly indicates the motility of CDs in solid-state PR, although the PR powder material used is not yet suitable for processing into various functional materials and devices.

In recent years, our group has developed a novel synthetic strategy for creating PR block copolymers by end-capping PPR with polymer chains via atom transfer radical polymerization (ATRP) [28,29,30,31,32,33,34]. This one-pot strategy not only significantly simplifies PR preparation but also leads to the formation of PR-based triblock copolymers, introducing new functionalities to solid PR materials through the incorporation of polymer chains. In our previous work, a PR-containing triblock copolymer was synthesized via ATRP of NIPPAAm, initiated by the self-assembly of α-cyclodextrins (α-CD) with distal 2-bromopropionyl end-capped poly(caprolactone) in water at 25 °C. Subsequently, azobenzene groups were introduced into the hydroxyl groups of threaded α-CD to obtain azo-substituted polyrotaxane, capable of forming micelles in aqueous solution. The incorporation of PNIPPAAm blocks as the end-capping group conferred thermal-responsive drug release behavior to the micelles, alongside photo-responsive release behavior from the azobenzene groups [31]. However, this dual responsiveness occurred in solution due to the hydrophilic PNIPPAAm block’s lack of mechanical strength.

In this paper, we employed hydrophobic polybutylmethacrylate (PBMA) chains to end-cap PPR formed by F68 (PEO-PPO-PEO copolyether) and β-CDs via the ATRP strategy, resulting in solid PR materials. The inclusion of the hydrophobic polymer chain imparts high mechanical strength to the PR materials. We explored the motility of threaded CDs in solid PR materials through dielectric measurements and thoroughly investigated the impact of highly mobile CDs on the mechanical properties of solid PR materials. This work lays a solid foundation for designing and developing diverse advantageous applications for solid PR materials in the future by combining and fully utilizing the motility of PR moieties and the robust mechanical properties of solid materials.

## 2. Materials and Methods

### 2.1. Materials

β-cyclodextrin (β-CD, China National Pharmaceutical Reagent Company, Shanghai, China), Pluronic F68 (Mn = 8400, molecular composition PEO70-PPO30-PEO70, Sigma-Aldrich, St. Louis, MO, USA). Cuprous chloride (Cu(I)Cl) was purified by stirring in acetic acid, washed with methanol, and finally dried under vacuum for use. 2-Bromopropionyl bromide (BrP, Alfa Aesar, Shanghai, China) and 4-dimethylaminopyridine (DMAP, Alfa Aesar, Shanghai, China), N,N-diethylethylamine (TEA, Alfa Aesar). N,N-dimethylformamide (DMF, chromatography grade, Beijing Tong Guang Fine Chemiacals Company, Beijing, China). All reagents and solvents are of analytical grade unless otherwise stated.

### 2.2. Methods

^1^H NMR (500 MHz) spectra were recorded on a Bruker Avance 3 HD spectrometer at room temperature and tetramethylsilane (TMS) as an internal standard. FTIR spectra were measured using a Shimadzu IR Prestige-21 FTIR spectrometer in the range between 4000 and 500 cm^−1^ with a resolution of 2 cm^−1^. The samples were dispersed in KBr, compressing the mixtures to form disks. Gel permeation chromatography (GPC) measurements were carried out at 40 °C on a TOSOH EcoSEC GPC instrument using DMF as eluent with a flow rate of 0.3 mL/min. LiBr (10 mmol/L) was added to the eluent to suppress the association.

Samples were cut into dumbbell shapes, with a sample area of 20 mm in length, 4 mm in width, and 0.1–0.2 mm in thickness, according to the international testing standard ISO 527-3:2018 [35]. This shape helps simulate the stress concentration scenarios that materials might encounter in real-world applications, allowing for a more accurate evaluation of the material’s mechanical properties. The tensile strength was measured by a tensile speed of 100 mm/min to test the tensile strength of the materials, and three sample strips were tested in parallel for each sample. The room temperature at the time of testing was 25 °C. The tensile properties were measured by a DLL-5000 electronic tensile test apparatus from Shanghai D&G Measuring Instrument Co., Ltd., Shanghai, China. The static mechanical analysis was carried out on the SHIMADZU TMA-60 with film tension mode, and the sample was cut into strips with a width of around 1 cm and a length of 10–12 cm. The dynamic mechanical analysis was tested with dumbbell-shaped samples on the RSA-III Dynamic Mechanical Analyzer. The dielectric measurement was carried out on the Alpha-S High Resolution Dielectric Analyzer with a ZGS Alpha Active Sample Cell, Novo Control, and the film sample was cut into a round shape by a mold.

### 2.3. Synthesis of Distal 2-Bromopropionyl End-Capped Pluronic F68(BrP-F68-PBr)

Pluronic F68 was converted to the corresponding ATRP macroinitiator by the end-capping reaction with a 4-fold molar excess of 2-bromopropionyl bromide in CH_2_Cl_2_ using a similar method as that described previously [31]. ^1^H NMR analysis was used to calculate the degree of esterification (>98%). ^1^H NMR (DMSO-d6): δ_4.7_ (CH_3_-C*H*Br-COO-), δ_4.23_ (-C*H_2_*-OOC-), δ_1.71–1.73_ (C*H_3_*-CHBr-) ppm.

### 2.4. Synthesis of PR-Based Triblock Copolymer (PR Copolymer, PBMA-b-PR-b-PBMA)

The BrP-F68-PBr (0.336 g, 0.04 mmol) was dissolved in 4 mL distilled water, while β-CD (0.68 g, 0.6 mmol) was dissolved in 12 mL water at 60 °C. After then, two solutions were mixed slowly and stirred for 24 h. The PPR initiator was precipitated gradually from the mixing solution and separated by centrifuge after reaction. The wet PPR initiator was dried by lyophilization.

The PPR initiator (0.522 g, 0.02 mmol), BMA (1.991 g, 14 mmol), and PMDETA (8.4 mg, 0.048 mmol) were added in a 15 mL Pyrex tube. The tube was frozen by liquid nitrogen and degassed with nitrogen thrice. Then, CuCl (7.96 mg, 0.08 mmol) was added into the tube, and the tube was sealed under vacuum. The tube was heated to 50 °C and kept for 16 h. The reaction was terminated by breaking the Pyrex tube. The crude product was first dissolved in the mixture of 30 mL DMSO and 20 mL THF and then placed in the dialysis tubing (MWCO = 3500) to dialyze against water for 24 h. The water was changed with an interval of 8 h. After drying by lyophilization, the solid was dissolved in 50 mL DMF again and dialyzed for another 24 h in order to remove the free β-CD and unreacted PPR. The solid was washed by 20 mL methanol and 30 mL water successively in order to remove unreacted BMA before it was finally dried by lyophilization. ^1^H NMR (DMSO-d_6_): δ_5.74_ (-O-C*H*-CH-O-), δ_4.88_ (-O-C*H*-O-), δ_4.42_ (-O-C*H*_2_-CH-O-), δ_3.94_ (-O-C*H*_2_-CH_2_-CH_2_-CH_3_), δ_3.64_ ((-C*H*_2_-O-*CH*_2_-*CH*_2_-O-), δ_3.40_ (-OC*H*CH_3_-CH_2_-O-), δ_2.05–1.70_ (-C*H*_2_-CH-CH_3_Br-CO-), δ1.60 (-C*H*_2_-CH_2_-CH_3_), δ_1.38_ (CH_3_-C*H*_2_-CH_2_-CH_2_-O-), δ_1.13_ (C*H_3_*-CH_2_-O-), δ_0.94_ (C*H*_3_-CH_2_-CH_2_-CH_2_-O-) ppm.

### 2.5. Preparation of F68 Copolymer (PBMA-b-F68-b-PBMA, Control Sample)

The macroinitiator BrP-F68-PBr (0.168 g, 0.02 mmol), BMA (1.991 g, 14 mmol), and PMDETA (8.4 mg, 0.048 mmol) were added in a 15 mL Pyrex tube. The tube was frozen by liquid nitrogen and degassed with nitrogen thrice. Then, CuCl (7.96 mg, 0.08 mmol) was added into the tube, and the tube was sealed under vacuum. The tube was heated to 50 °C and kept for 16 h. The reaction was terminated by breaking the Pyrex tube. The crude product was first dissolved in 20 mL CHCl_3_ and precipitated in 100 mL methanol. After three dissolving–precipitating cycles, the product was obtained and dried in a vacuum oven for 24 h (yield = 81.2%). ^1^H NMR (CDCl_3_-d_1_): δ_3.94_ (-O-C*H*_2_-CH_2_-CH_2_-CH_3_), δ_3.64_ (-C*H*_2_-O-*CH*_2_-*CH*_2_-O-), δ_3.40_ (-OC*H*-CH_3_-CH_2_-O-), δ_2.05–1.70_ (-C*H*_2_-CHCH_3_Br-CO-), δ_1.60_ (-C*H*_2_-CH_2_-CH_3_), δ_1.38_ (CH_3_-C*H*_2_-CH_2_-CH_2_-O-), δ_1.13_ (C*H*_3_-CH-O-), δ_0.94_ (C*H_3_*-CH_2_-CH_2_-CH_2_-O-) ppm.

### 2.6. Preparation of PR and F68 Copolymers Films

The films were prepared by the casting method. Approximately 0.73 g sample was dissolved in 30 mL of chloroform, pouring them into a PTFE mold, and then letting them stand for 12 h to allow the solvent to evaporate in order to make the films. After completion, the sample films were dried under vacuum at 25 °C for 12 h to remove the solvent to obtain the sample films.

### 2.7. Statistical Analysis

Statistical analysis of quantitative data from tensile tests was performed on three parallel samples. Statistical analysis between the two groups was performed by Student *t*-test using SPSS (version 26; IBM, Armonk, NY, USA), where * *p* < 0.05, ** *p* < 0.01 and *** *p* < 0.001.

## 3. Results and Discussion

### 3.1. Preparation and Characterization of PR Copolymer

**Scheme 1:**
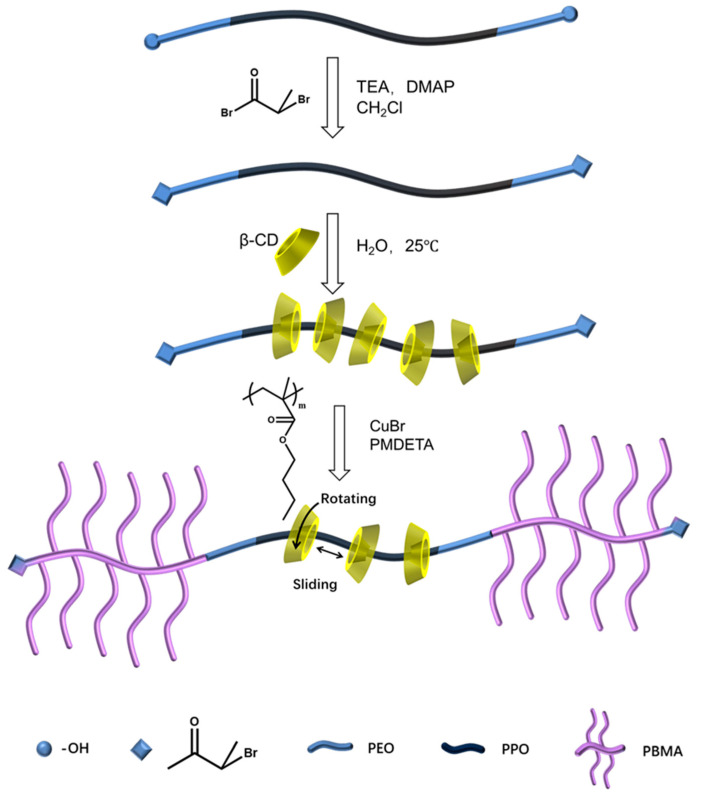
The synthetic route of PR copolymer.

The synthetic route for the PR copolymer by ATRP is illustrated in Scheme 1 and Figure S1. The PPR initiator was prepared through the self-assembly of β-CD and BrP-F68-PBr in an aqueous solution. Cu(I)Cl is the catalyst in the ATRP, but it is easy to be oxidized to CuCl_2_ by O_2_ in the air to lose the catalytic activity. Thus, it should be purified before use. In our previous studies, we successfully conducted ATRP reactions of hydrophilic vinyl monomers such as HEMA [34], NIPPAAm [33,36], and PEGMA [29,37] in situ to end-cap PPR, resulting in PR triblock polymers. This approach involved a one-pot strategy. However, this method is not suitable for our current needs since BMA is hydrophobic. Therefore, we adopted a two-step strategy for preparing the PR copolymer. In the first step, after forming the PPR initiator, it was separated from the aqueous solution by lyophilization. Subsequently, in the second step, bulk ATRP of BMA was performed using the PPR initiator to synthesize the PR copolymer.

The synthetic results for both the PR copolymer and the control sample, F68 copolymer, are presented in Table 1. The degree of polymerization (DP) of BMA in both copolymers was closely maintained. However, a characteristic of ATRP reactions is the precise control of DP through feed ratio adjustments. This means that the primary difference between the PR copolymer and the F68 copolymer lies solely in the composition and structure of their middle blocks. The PR copolymer features a middle block composed of PR units formed by F68 and threaded β-CDs, whereas the F68 copolymer comprises a middle block solely made from F68 units without threaded β-CDs. Therefore, it would be convenient and insightful to compare the dielectric and mechanical properties of these two triblock copolymers and explore the influence of the rotaxane structure on the PR copolymer.

In Table 1, it is observed that the number of threaded β-CDs in the PR copolymer is significantly fewer than the initial feed ratio. This reduction may be attributed to the dethreading of β-CDs during the bulk ATRP process, a phenomenon documented in the literature. Despite this, the rotaxane structure, albeit with fewer threaded β-CDs, remains intact in the PR copolymer. This retained rotaxane structure provides a unique opportunity to investigate how it influences the dielectric and mechanical properties of solid PR materials. By studying these influences, we can gain insights into how the presence of the rotaxane structure affects the overall performance and characteristics of the PR copolymer compared to copolymers without threaded β-CDs. This exploration is crucial for understanding the potential applications and functional properties that the rotaxane structure imparts to these materials.

### 3.2. The Characterization of PR Copolymer

**Figure 1:**
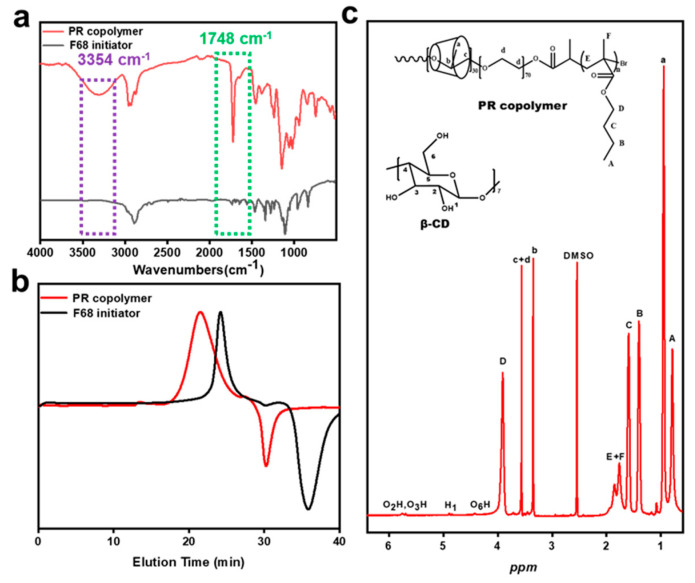
Graphical characterization of PR copolymer and F68 initiator (**a**) FT-IR, (**b**) GPC, (**c**) ^1^H NMR.

The IR spectrum is shown in Figure 1a. Compared to the F68 macroinitiator, the presence of a hydroxyl absorption peak at 3354 cm^−1^ in PR’s spectrum indicates the presence of CDs, while the carboxyl absorption peak at 1748 cm^−1^ suggests successful preparation of the PR copolymer through ATRP of BMA. The GPC curves of F68 macroinitiator and PR copolymer are shown in Figure 1b. The curve of PR significantly shifts to the left compared to that of the F68 macroinitiator, indicating an increase in molecular weight of the PR copolymer due to BMA’s ATRP. Furthermore, the PR curve displays a single, symmetric peak without any side peaks, which confirms the successful preparation of the PR copolymer. The NMR spectrum, displayed in Figure 1c, clearly shows all marked resonance peaks. The resonance peaks at δ = 5.5, 4.8, and 4.5 ppm were assigned to the protons O_2_/O_3_, H_1_, and O_6_ of the β-CD moiety, respectively. The other characteristic peaks of the β-CD moiety, ranging from 3.0 to 4.0 ppm, overlapped with those of the F68 and PBMA moieties in the PR copolymer. Thus, the occurrence of O_2_/O_3_, H_1_, and O_6_ resonance peaks preliminarily suggests the presence of threaded β-CDs in the PR copolymer. Furthermore, the presence of PBMA’s resonance peaks (A~F) suggests successful end-capping via the ATRP reaction. Additionally, the resonance peaks (a–d) corresponding to the F68 moiety were also present in the PR copolymer’s NMR spectrum. Since all blocks, including β-CD, F68, and PBMA, were identifiable in the NMR spectrum, this indicates the successful preparation of the PR copolymer. Furthermore, the GPC curve did not show any side peaks, implying the absence of free β-CDs in the as-prepared PR copolymer. It can be concluded that the β-CDs are threaded onto the F68 main chain, forming a mechanically interlocked rotaxane structure. Therefore, the IR, NMR, and GPC results collectively demonstrate the successful synthesis of the PR copolymer via ATRP of BMA. The number of threaded β-CDs (n) in the PR copolymer can be calculated from the NMR spectrum using Equation (1).

n = [3 × 30 × A_4.8(H1)_] ÷ 7A_3.5(b)_ (1)

wherein Ax represents the integrated area under x ppm, and the results are shown in Table 1. The number of threaded CDs was calculated as 3.5, thus the prepared PR copolymer possessed low CD coverage as the full coverage should be 15 for one F68 molecule. Furthermore, the DP of PBMA was calculated according to the following Equation (2), and the results are listed in Table 1.

DP = [3 × 30 × A_4.0(D)_]/7A_3.5(b)_ (2)

### 3.3. The Motility of Threaded CDs in PR Copolymers

**Figure 2:**
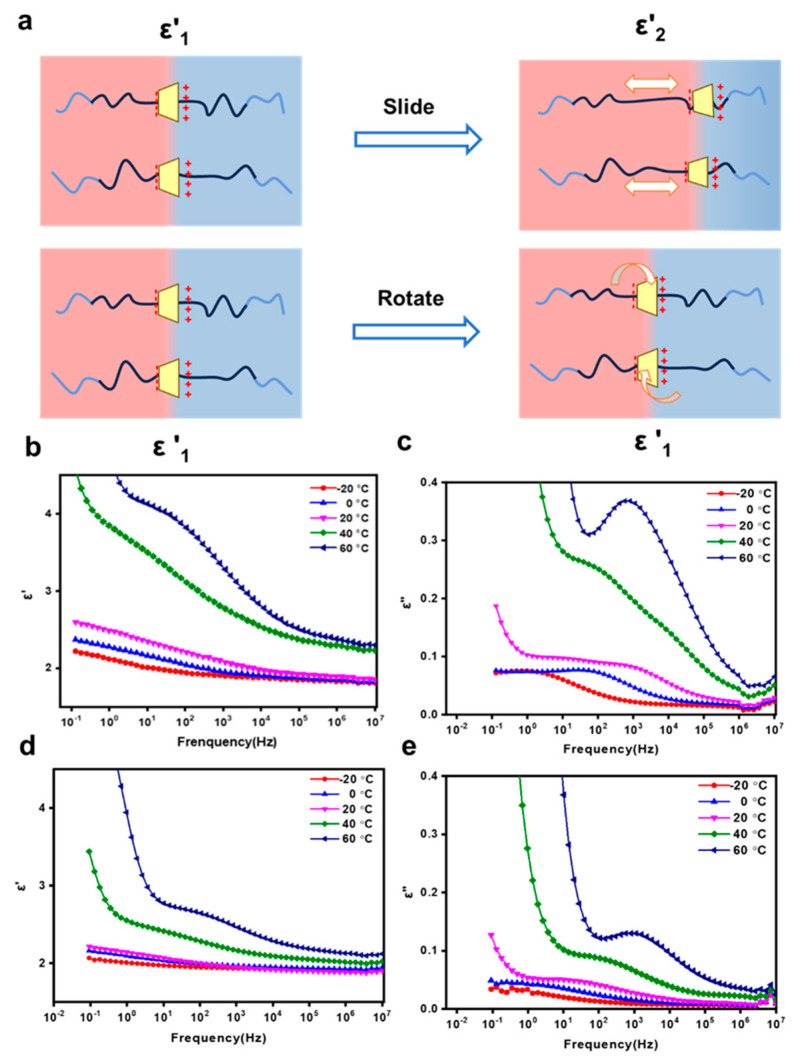
The dielectric spectra of PR and F68 copolymers. (**a**) Diagram of the influence of CD motion on the dielectric property, (**b**) the real part of PR copolymer’s dielectric spectrum, (**c**) the imaginary part of PR copolymer’s dielectric spectrum, (**d**) the real part of F68 copolymer’s dielectric spectrum, (**e**) the imaginary part of F68 copolymer’s dielectric spectrum.

**Figure 3:**
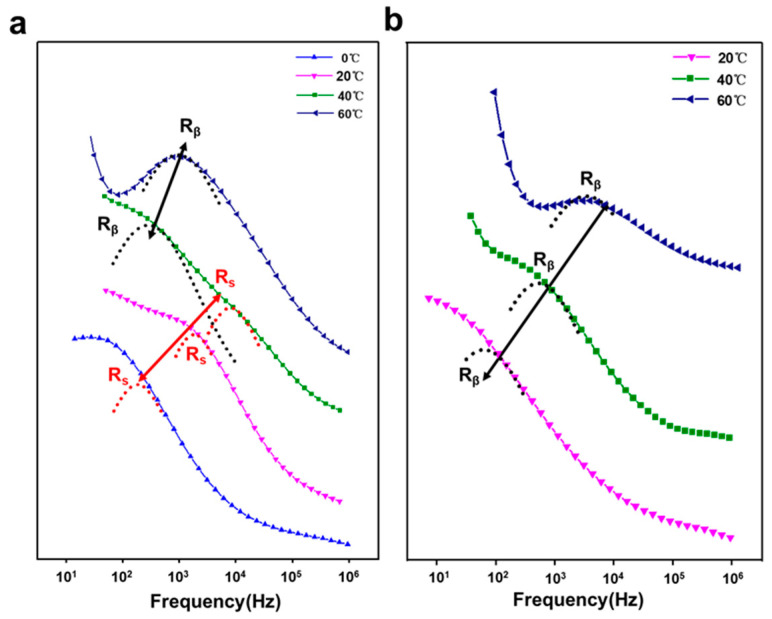
(**a**) The imaginary part of PR copolymer’s dielectric spectrum; (**b**) imaginary part of the peak plot of the dielectric spectrum of F68 copolymer.

To observe the molecular mobility of β-CD on the PR copolymer, dielectric spectroscopy was conducted on both PR and F68 copolymers, with results presented in Figure 2. Due to strong electrode polarization at low frequencies, dielectric constants sharply increased and were less reliable in the low-frequency range for both the real and imaginary parts. However, analysis of the imaginary part reveals distinct differences: the F68 copolymer exhibits a single relaxation process (Rβ) in the frequency range of 10^−1^ to 10^4^ Hz at temperatures ranging from 0 to 60 °C, attributed to the β-relaxation of the PBMA block as reported in the literature [38,39]. In contrast, the dielectric spectra of the PR copolymer display a flattened arc pattern rather than a single peak (Figure 2c), suggesting the presence of additional relaxation processes. Upon peak separation using software, another relaxation process (Rs) emerges clearly between 100 and 105 Hz at temperatures from −20 to 60 °C in the imaginary part of the PR copolymer’s dielectric spectra in Figure 3a. Given the molecular structural differences between PR and F68 copolymers, this new relaxation peak is attributed to the motion of β-CDs within the PR block. Similar relaxation processes have been observed in PR powder samples by Ito and colleagues, where bulky organic compounds were used for end-capping [38,39]. β-CDs in the PR block exhibit two modes of motion: sliding along and rotating around the F68 axis. However, rotational motion of β-CDs does not induce corresponding dielectric relaxation due to negligible changes in dipole moment, as shown in Figure 2a. Therefore, the observed relaxation peaks are linked to the sliding motion of β-CDs along the F68 chain segment. This sliding motion contributes to dielectric relaxation through changes in dipole moment direction and localized modal or micro-Brownian motions of F68 associated with immobilized β-CD dipole moments on the chain.

Therefore, these results demonstrate that the movement of β-CDs occurs in solid PR materials, not just in solution or powder samples. This suggests that the advantageous properties of PR observed in solution and powder states may similarly manifest in solid PR materials. This insight could encourage materials researchers to design and develop various solid PR materials with enhanced performance. Furthermore, after proving the motility of β-CDs by the dielectric measurement, further research can be conducted on the influence of β-CD’s motility on the mechanical properties of PR copolymers.

### 3.4. The Influence of CD’s Motility on Mechanical Properties of PR Copolymers

**Figure 4:**
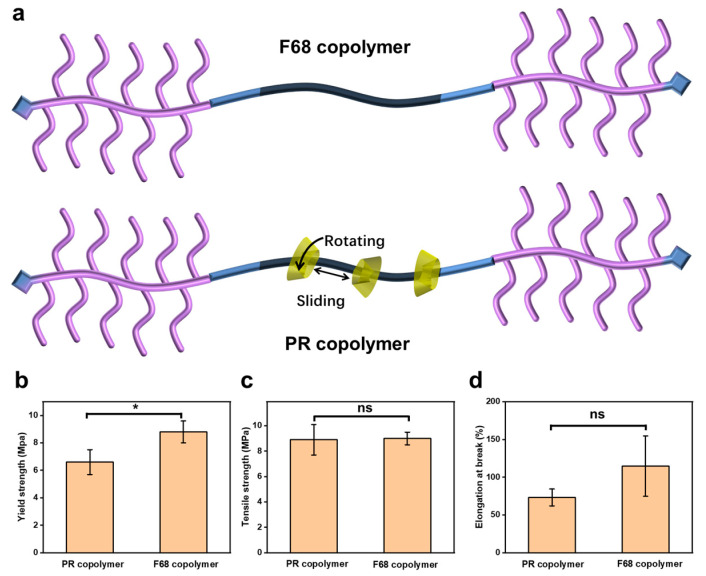
Mechanical strength results of PR and F68 copolymer. (**a**) Chemical structure of F68 and PR copolymers, (**b**) yield strength, (**c**) tensile strength, (**d**) elongation at break.

To investigate the influence of β-CDs’ motion on the mechanical properties of PR material, we prepared F68 copolymers without any threaded β-CDs as the control group. As shown in Figure 4a, the key difference between the PR copolymer and the F68 copolymer is that the PR copolymer possesses highly mobile β-CDs threaded on the main chain. This allows us to understand the influence of the mobility of CDs on the mechanical properties of PR copolymers by correlating the different performances of PR and F68 copolymers in various mechanical measurements with their structural differences. Tensile tests were conducted on PR and F68 copolymer materials. The results are summarized in Table 1 and depicted in Figure 4b–d. It was observed that PR and F68 copolymers exhibit similar tensile strengths without statistical difference. This similarity arises primarily because the mechanical strength of both PR and F68 copolymers is predominantly governed by the PBMA block. However, distinct differences were found in yield strength, as shown in Table 1. The yield strength of PR copolymers was significantly lower than that of F68 copolymers in statistic. Since both PR and F68 copolymers possess similar PBMA blocks at both ends, the differences in yield strength can be mainly attributed to variations in the two middle blocks. DSC measurements (Figure S5) have demonstrated that the mobility of threaded CDs suppresses the crystallization of the PR block in the PR copolymer, which likely contributes to its lower yield strength.

**Figure 5:**
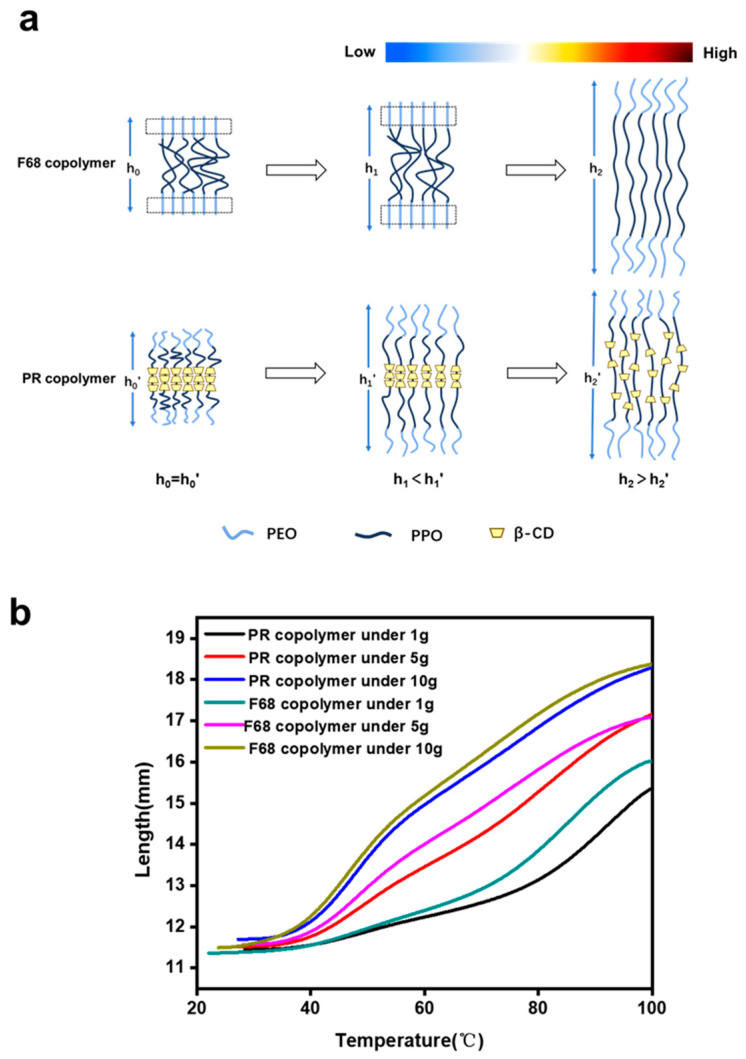
(**a**) Diagram of the influence of CD motion on the thermal-mechanical behavior; (**b**) the TMA curves of PR and F68 copolymers.

Thermal mechanical analysis was conducted under three different tensions, showing a consistent trend as depicted in Figure 5b. Firstly, it is evident that higher tensions result in larger deformations. Secondly, more pronounced differences were observed under the smallest tension. The molecular mechanism responsible for this difference is shown in Figure 5a. In the first stage, characterized by temperatures below 40 °C, both PR and F68 copolymers exhibited minimal deformation, primarily due to elastic deformation of the copolymers. The deformation of PR copolymer is slightly larger than F68 copolymer, due to the absence of crystalline structure in the middle PR block resulting from the threaded CDs. Moving to the second stage (40 °C to 60 °C), temperatures exceeded the glass transition temperature (T_g_) of the PBMA blocks, allowing segments of the PBMA blocks to undergo more significant movement. This resulted in increased deformation for both PR and F68 copolymers compared to the first stage. In the third stage (above 60 °C), intermolecular interactions weakened further, and the F68 blocks in both copolymers began to soften and approach melting, leading to a sharp increase in deformation.

However, in the PR copolymer, the movement of β-CDs likely “shielded” the F68 chain within the PR block to some extent, unlike the bare F68 block in the F68 copolymer. This shielding effect may have prevented the F68 chain in the PR block from softening and melting as much as in the F68 copolymer, resulting in noticeably smaller deformation in the PR copolymer compared to the F68 copolymer in the third stage. Therefore, the presence of β-CDs appears to play an important role in modulating the thermal mechanical properties of the PR copolymer, providing it with enhanced stability and reduced deformation at higher temperatures compared to the F68 copolymer.

**Figure 6:**
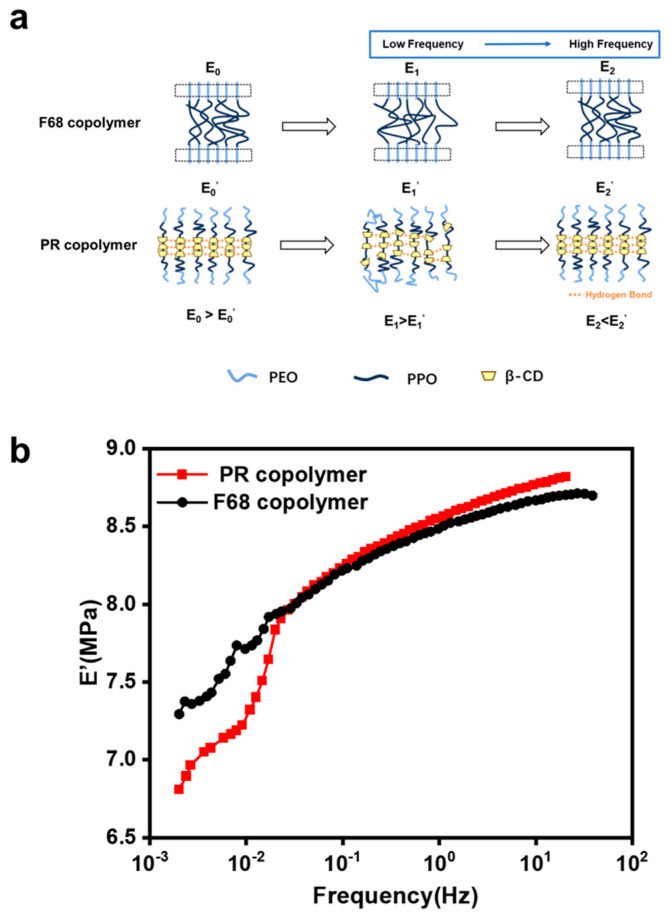
(**a**) Diagram of the influence of CD motion on the dynamic-mechanical behavior; (**b**) the storge moduli of PR and F68 copolymers measured by DMA.

The results of the dynamic mechanical analysis (DMA) are presented in Figure 6b. The data indicate that the storage modulus of the PR copolymer is lower than that of the F68 copolymer in the low-frequency range, while it is higher in the high-frequency range. The molecular mechanisms responsible for these differences in the DMA curves are shown in Figure 6a. In the low-frequency range, the motion of β-CDs can keep up with the periodic changes of external force. This allows the PR copolymer to respond in a timely manner to external forces through the relative motion of threaded CDs and F68 segments, resulting in a relatively low modulus. In contrast, the F68 copolymer, with its crystalline structure of PEO segments and intermolecular random coil-like entanglement, has more difficulty responding to changes in external forces. In the high-frequency range, the motion of β-CDs cannot keep up with the periodic changes of external force. Consequently, the PR copolymer cannot respond to external forces through the motion of threaded β-CDs. At this point, the PR copolymer exhibits a higher modulus than the F68 copolymer due to intermolecular hydrogen bonds between threaded β-CDs, which enhance the mechanical strength of the PR copolymer. In conclusion, the PR copolymer and the F68 copolymer exhibit distinct mechanical properties, likely due to the movement of threaded β-CDs in the PR copolymer. The observed differences in mechanical properties, as evidenced by tensile, TMA, and DMA tests, along with dielectric measurements, suggest that the molecular motility of β-CDs significantly influences the mechanical properties of PR materials.

In summary, the significant differences in the mechanical properties of the two copolymers are attributed to the motility of β-CDs in the PR block. These mechanical property results, together with dielectric measurements, not only demonstrate that threaded β-CDs are moveable in solid PR materials but also indicate that their motility can influence the macroscopic properties of these materials. Furthermore, it is important to note that other outstanding properties, besides mechanical ones, can also be expected for solid PR materials due to the motility of β-CDs. The mobility of β-CDs offers various valuable potential applications that are worth investigating in future research. For instance, the literature has proven that highly mobile threaded CDs can regulate the spreading and proliferation of endothelial cells. Therefore, the as-prepared PR copolymer may be used to promote the angiogenesis of porous scaffolds in tissue engineering [17].

However, there are some limitations in this study. First, it did not involve the regulation of the number of threaded β-CDs. As mentioned above, the number of threaded β-CDs is only 3.5 per chain, which is far from full coverage (15 β-CDs per chain). Increasing the number of threaded β-CDs may decrease their motility on the main chain but could enhance the intermolecular hydrogen bonds. Therefore, the influence of cyclodextrin quantity on the mechanical properties of materials is worth studying. In our future work, we will further optimize the preparation protocol to obtain PR copolymer with a controlled number of threaded β-CDs, allowing for a more detailed investigation of their effects on mechanical properties. Second, this study only involved the self-assembly of F68’s PPO block and β-CDs. It is well known that F68’s PEO blocks can self-assemble with α-CDs. However, the influences of mobile threaded α-CDs on the mechanical properties of PR copolymers are still unclear, which is a very interesting aspect to be investigated in future studies.

## 4. Conclusions

The PR copolymer was successfully synthesized via bulk ATRP reaction of BMA, initiated with PPRs comprising bromo-terminated F68 and β-CDs, in the presence of Cu(I)Br/PMDETA. This copolymer can form a self-standing film, and the motility of β-CDs in the solid PR material was confirmed by dielectric measurements. Compared with the control sample F68 copolymer without mobile β-CDs, the PR copolymer exhibits lower yield strength, higher strain at low temperatures, lower strain at high temperatures, lower modulus at low frequencies, and higher modulus at high frequencies. These results demonstrated that the mobility of threaded β-CDs significantly influences the mechanical properties of the PR copolymer. Compared with the F68 copolymer, the PR copolymer exhibits lower yield strength and lower modulus at low frequencies while maintaining nearly the same tensile strength. This demonstrates the potential application of PR copolymer as a high-toughness material in the future. Additionally, the motility of β-CDs might provide other outstanding properties beyond mechanical ones, so as to offer various potential applications for PR copolymers, for instance, to amplify the biological functions of ligand grafted on the mobile CDs.

## Figures and Tables

**Table 1:** The synthetic and stress-stain measurement results of PR and F68 copolymers.

Name	Molar Composition F68:β-CD:BMA		Molecular Weight and Polydispersity Index			Yield /%	Tensile Strength/MPa	Elongation at Break/%	Yield Strength/Mpa
	Feed Ratio	Found Ratio ^a^	M_n_’ 10^3 a^	M_n_ × 10^3 b^	PDI				
PR copolymer	1:15:700	1:3.5:787	125	87	1.14	55.2	12.3 ± 1.7	73.1 ± 11	6.6 ± 0.9
F68 copolymer	1:700	1:738	119	76	1.21	63.7	11.9 ± 1.3	114.7 ± 39	8.8 ± 0.8
F68 initiator	N/A	N/A	43	40	1.29	50.2	N/A	N/A	N/A

^a^ Calculated from 1H-NMRspetrum. ^b^ Obtained from GPC measurement and calibrated by PS standard sample.

## Data Availability

The original contributions presented in the study are included in the article/Supplementary Materials, further inquiries can be directed to the corresponding author.

## Supplementary Materials

The following supporting information can be downloaded at: https://www.mdpi.com/article/10.3390/ma17153757/s1, Figure S1: Synthesis route of PR copolymer; Figure S2: FT-IR spectra of F68 copolymers; Figure S3: 1H-NMR spectrum of F68 copolymer after capping; Figure S4: The GPC traces of F68 copolymer; Figure S5: The DSC curves of PR and F68 copolymers.

## References

[1] Du R., Bao T., Kong D., Zhang Q., Jia X. (2024). Cyclodextrins-Based Polyrotaxanes: From Functional Polymers to Applications in Electronics and Energy Storage Materials. ChemPlusChem.

[2] Higashi T., Taharabaru T., Motoyama K. (2024). Synthesis of cyclodextrin-based polyrotaxanes and polycatenanes for supramolecular pharmaceutical sciences. Carbohydr. Polym..

[3] Chen L., Sheng X., Li G., Huang F. (2022). Mechanically interlocked polymers based on rotaxanes. Chem. Soc. Rev..

[4] Harada A. (2001). Cyclodextrin-based molecular machines. Acc. Chem. Res..

[5] Zhang P., Qian X., Zhang Z., Li C., Xie C., Wu W., Jiang X. (2017). Supramolecular amphiphilic polymer-based micelles with seven-armed polyoxazoline coating for drug delivery. ACS Appl. Mater. Interfaces.

[6] Cui Q., Zhang X. (2023). Polyrotaxane-Based Functional Materials Enabled by Molecular Mobility and Conformational Transition. Chin. J. Chem..

[7] Masuda H., Arisaka Y., Hakariya M., Iwata T., Yoda T., Yui N. (2023). Molecular Mobility of Polyrotaxane Surfaces Alleviates Oxidative Stress-Induced Senescence in Mesenchymal Stem Cells. Macromol. Biosci..

[8] Arisaka Y., Yui N. (2019). Polyrotaxane-based biointerfaces with dynamic biomaterial functions. J. Mater. Chem. B.

[9] Mayumi K., Liu C., Yasuda Y., Ito K. (2021). Softness, elasticity, and toughness of polymer networks with slide-ring cross-links. Gels.

[10] Liu C., Yokoyama H., Mayumi K., Ito K. (2021). Crack velocity dependent toughness of polyrotaxane networks: The sliding dynamics of rings on polymer under stretching. Mech. Mater..

[11] Liu C., Morimoto N., Jiang L., Kawahara S., Noritomi T., Yokoyama H., Mayumi K., Ito K. (2021). Tough hydrogels with rapid self-reinforcement. Science.

[12] Liu S., Hayashi T., Hara M., Seki T., Ito K., Takeoka Y. (2024). Optimal conditions for the use of polyrotaxane as a cross-linker in preparing elastomers with high toughnesses. Polym. J..

[13] Huang J., Ren L., Chen Y. (2008). pH-/temperature-sensitive supramolecular micelles based on cyclodextrin polyrotaxane. Polym. Int..

[14] Han Z., Zhou Q., Li Y. (2018). Self-assembled (pseudo) rotaxane and polyrotaxane through host–guest chemistry based on the cucurbituril family. J. Incl. Phenom. Macrocycl. Chem..

[15] Seo J.H., Yui N. (2013). The effect of molecular mobility of supramolecular polymer surfaces on fibroblast adhesion. Biomaterials.

[16] Arisaka Y., Masuda H., Yoda T., Yui N. (2021). Delayed senescence of human vascular endothelial cells by molecular mobility of supramolecular biointerfaces. Macromol. Biosci..

[17] Zhong X., Zhang S., Wang H., Wang M., Feng Z., Su W., Wang J., Liu Z., Ye L. (2024). Dynamic RGD ligands derived from highly mobile cyclodextrins regulate spreading and proliferation of endothelial cells to promote vasculogenesis. Int. J. Biol. Macromol..

[18] Sekiya-Aoyama R., Arisaka Y., Yui N. (2020). Mobility tuning of polyrotaxane surfaces to stimulate myocyte differentiation. Macromol. Biosci..

[19] Liu Z., Ye L., Xi J., Wang J., Feng Z.-G. (2021). Cyclodextrin polymers: Structure, synthesis, and use as drug carriers. Prog. Polym. Sci..

[20] Arunachalam M., Gibson H.W. (2014). Recent developments in polypseudorotaxanes and polyrotaxanes. Prog. Polym. Sci..

[21] Tu C., Zhang Y., Xiao Y., Xing Y., Jiao Y., Geng X., Zhang A., Ye L., Gu Y., Feng Z. (2022). Hydrogel-complexed small-diameter vascular graft loaded with tissue-specific vascular extracellular matrix components used for tissue engineering. Biomater. Adv..

[22] Geng X., Xu Z.Q., Tu C.Z., Peng J., Jin X., Ye L., Zhang A.Y., Gu Y.Q., Feng Z.G. (2021). Hydrogel complex electrospun scaffolds and their multiple functions in in situ vascular tissue engineering. ACS Appl. Bio Mater..

[23] Ye L., Takagi T., Tu C., Hagiwara A., Geng X., Feng Z. (2021). The performance of heparin modified poly (ε-caprolactone) small diameter tissue engineering vascular graft in canine—A long-term pilot experiment in vivo. J. Biomed. Mater. Res. Part A.

[24] Duan N., Geng X., Ye L., Zhang A., Feng Z., Guo L., Gu Y. (2016). A vascular tissue engineering scaffold with core–shell structured nano-fibers formed by coaxial electrospinning and its biocompatibility evaluation. Biomed. Mater..

[25] Inomata A., Ishibashi H., Nakajima T., Sakai Y., Kidowaki M., Shimomura T., Ito K. (2007). Dielectric relaxation of liquid-crystalline polyrotaxane. Europhys. Lett..

[26] Inomata A., Kidowaki M., Sakai Y., Yokoyama H., Ito K. (2011). Orientational motions in mesogenic polyrotaxane and local mode relaxations of polymer segments in solid state polyrotaxane. Soft Matter.

[27] Inomata A., Sakai Y., Zhao C., Ruslim C., Shinohara Y., Yokoyama H., Amemiya Y., Ito K. (2010). Crystallinity and Cooperative Motions of Cyclic Molecules in Partially Threaded Solid-State Polyrotaxanes. Macromolecules.

[28] Jiang R.R., Kong T., Ye L., Zhang A.Y., Feng Z.G. (2015). Preparation of beta-Cyclodextrin-based Polyrotaxane Block Copolymers and Their Solvent-responsibily. Acta Polym. Sin..

[29] Jiang L., Gao Z.M., Ye L., Zhang A.Y., Feng Z.G. (2014). A Polyrotaxane-based pH-labile Drug Delivery System. Period. Polytech. Chem. Eng..

[30] Tong X., Gao P., Zhang X., Ye L., Zhang A.Y., Feng Z.G. (2010). End-capping double-chain stranded polypseudorotaxanes using lengthily tunable poly (2-hydroxyethyl methacrylate) blocks via atom transfer radical polymerization. Polym. Int..

[31] Wang P.J., Wang J., Ye L., Zhang A.Y., Feng Z.G. (2012). Synthesis and characterization of polyrotaxanes comprising α-cyclodextrins and poly (ε-caprolactone) end-capped with poly (N-isopropylacrylamide) s. Polymer.

[32] Lin J., Kong T., Ye L., Zhang A.Y., Feng Z.G. (2015). Self-assemblies of γ-CDs with pentablock copolymers PMA-PPO-PEO-PPO-PMA and endcapping via atom transfer radical polymerization of 2-methacryloyloxyethyl phosphorylcholine. Beilstein J. Org. Chem..

[33] Li S., Wang J., Gao P., Ye L., Zhang A., Feng Z.G. (2012). Polyrotaxane-based triblock copolymers synthesized via ATRP of N-isopropylacrylamide initiated from the terminals of polypseudorotaxane of Br end-capped pluronic 17R4 and β-cyclodextrins. Sci. China Chem..

[34] Tong X., Zhang X., Ye L., Zhang A.Y., Feng Z.G. (2009). Synthesis and characterization of block copolymers comprising a polyrotaxane middle block flanked by two brush-like PCL blocks. Soft Matter.

[35] (2018). Plastics—Determination of Tensile Properties: Part 3: Test Conditions for Films and Sheets.

[36] Wang J., Gao P., Ye L., Zhang A.Y., Feng Z.G. (2011). Dual thermo-responsive polyrotaxane-based triblock copolymers synthesized via ATRP of N-isopropylacrylamide initiated with self-assemblies of Br end-capped Pluronic F127 with β-cyclodextrins. Polym. Chem..

[37] Jiang L., Gao Z.M., Ye L., Zhang A.Y., Feng Z.G. (2013). A tumor-targeting nano doxorubicin delivery system built from amphiphilic polyrotaxane-based block copolymers. Polymer.

[38] Bergman R., Alvarez F., Alegrıa A., Colmenero J. (1998). Dielectric relaxation in PMMA revisited. J. Non-Cryst. Solids.

[39] Yan Z., Ye L., Zhang A.Y., Feng Z.G. (2017). The mobility of threaded α-cyclodextrins in PR copolymer and its influences on mechanical properties. Chin. J. Polym. Sci..

