# Supplementary material for: The Motility of β-Cyclodextrins Threaded on the Polyrotaxane Based Triblock Polymer and Its Influences on Mechanical Properties
Source: Materials (Basel). 2024 Jul 30;17(15):3757. doi: 10.3390/ma17153757 (PMC11313138; doi:10.3390/ma17153757)
Supplement: Supplementary file 1 [file materials-17-03757-s001.zip › materials-3121210-supplementary.pdf]

Supporting information

The Motility of β-Cyclodextrins Threaded on the Polyrotaxane Based Triblock Polymer and Its Influences on Mechanical Properties

Yufei Wang[1,2,†], Yafang Niu[1,2,†], Xue Geng[2], Zengguo Feng[2], Lin Ye[1,2,*]

1. Tangshan Research Institute, Beijing Institute of Technology, Tangshan 063000, China

2. School of Materials Science and Engineering, Beijing Institute of Technology, Beijing 100081, China

*Correspondence: yelin@bit.edu.cn

† These authors contributed equally to this work.

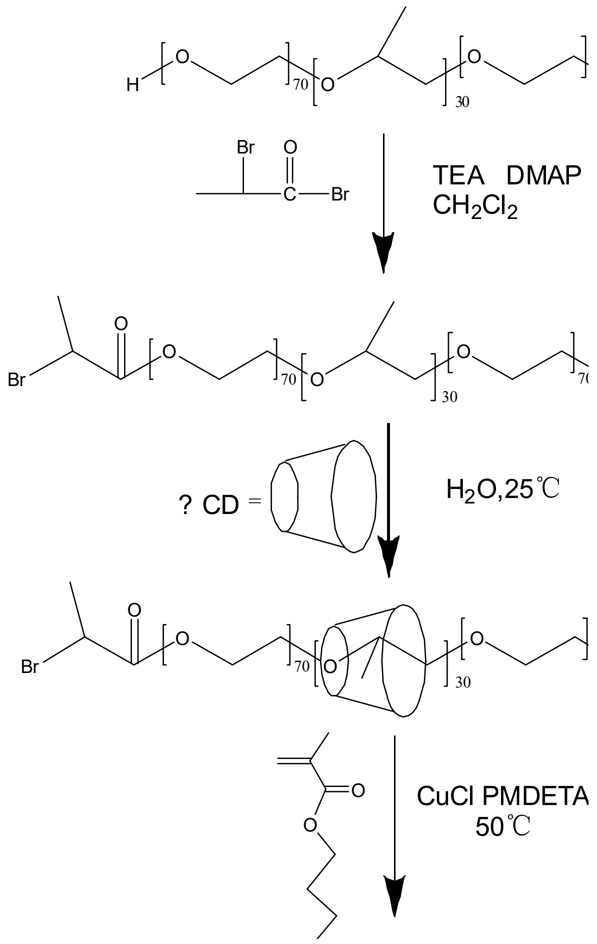

Figure S1. Synthesis route of PR copolymer

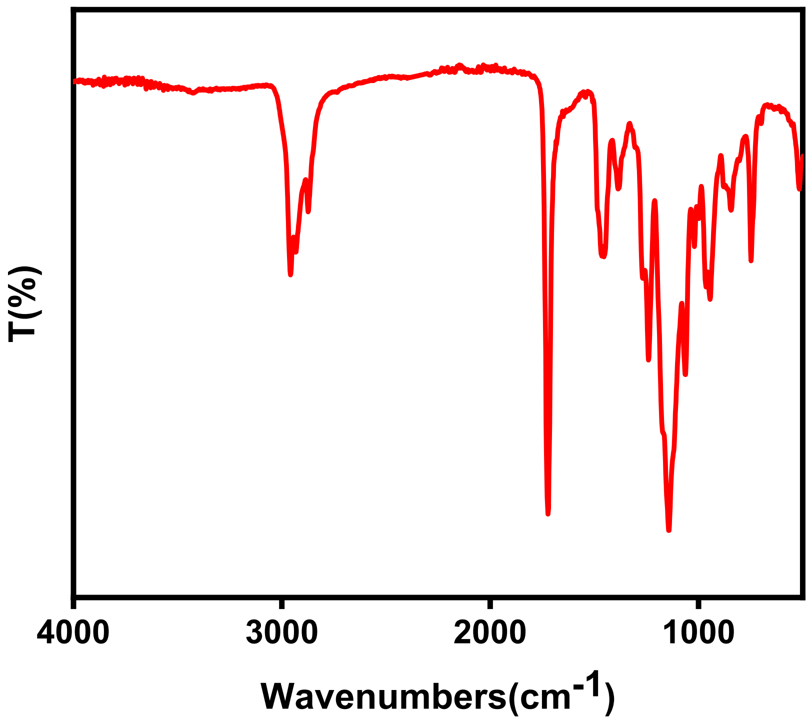

Figure S2. FT-IR spectra of F68 copolymers

The IR spectra are presented in Figure S2. The carboxyl absorption peak at 1748 cm^{-1} of the F68 copolymer provides evidence that the F68 copolymer was successfully prepared by ATRP of BMA. Furthermore, no hydroxyl absorption peak at 3354 cm^{-1} in the IR spectrum of the F68 copolymer was observed in comparison to the PR copolymer.

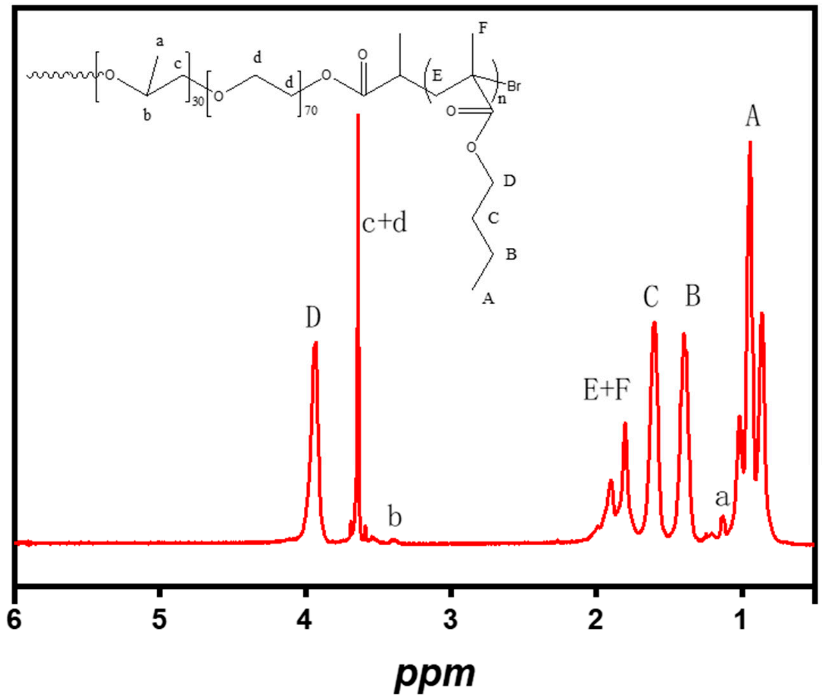

Figure S3. 1H-NMR spectrum of F68 copolymer after capping

Figure S3 showed the characteristic peaks of methyl (peak a) and methylene (peak b) in the PPO moiety were at 1.0 ppm and 3.3 ppm, while the characteristic peaks (A~F) of PBMA occurred in the spectrum. Thus, the successful preparation of F68 copolymer was demonstrated.

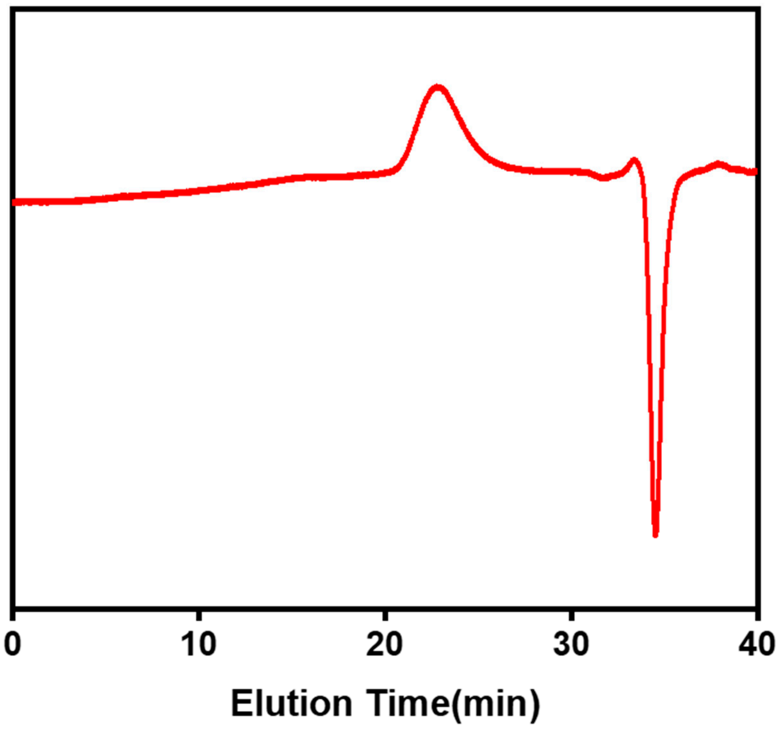

Figure S4. The GPC traces of F68 copolymer

Figure S4 showed the GPC traces of F68 copolymer. There is a single and symmetrical peak. This also implied the successful end-capping reaction via bulk ATRP of BMA.

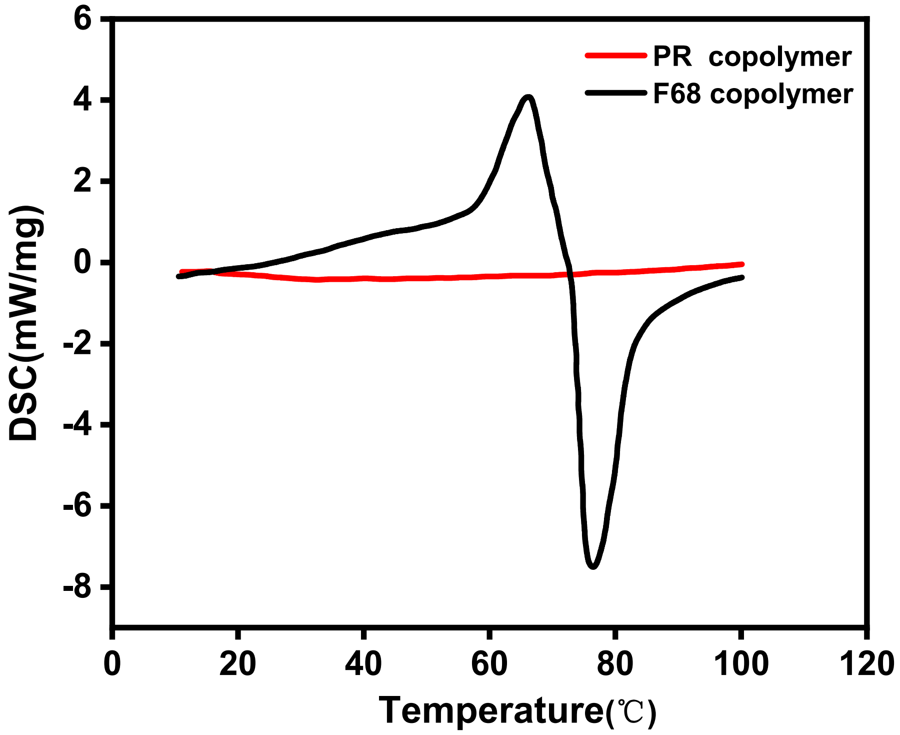

Figure S5. The DSC curves of PR and F68 copolymers

The F68 copolymer showed a clear melting peak of F68 crystallization around 76°C, but the PR copolymer sample did not show a clear melting peak of crystallization in the tested temperature range. It can be assumed that the addition of β-CD suppresses the crystallization of F68 in the original structure.
